# Diagnosis and Treatment of Urticaria and Angioedema: A Worldwide Perspective

**DOI:** 10.1097/WOX.0b013e3182758d6c

**Published:** 2012-11-15

**Authors:** Mario Sánchez-Borges, Riccardo Asero, Ignacio J Ansotegui, Ilaria Baiardini, Jonathan A Bernstein, G Walter Canonica, Richard Gower, David A Kahn, Allen P Kaplan, Connie Katelaris, Marcus Maurer, Hae Sim Park, Paul Potter, Sarbjit Saini, Paolo Tassinari, Alberto Tedeschi, Young Min Ye, Torsten Zuberbier, the WAO Scientific and Clinical Issues Council

**Affiliations:** 1 Department of Allergy and Clinical Immunology, Centro Médico-Docente La Trinidad, Caracas, Venezuela; 2 Ambulatorio di Allergologia, Clinica San Carlo, Paderno-Dugnano, Milan, Italy; 3 Department of Allergy and Immunology, Hospital Quirón Bizkaia, Bilbao, Spain; 4 Allergy and Respiratory Disease Clinic, University of Genova, Ospedale S.Martino di Genova, Genoa, Italy; 5 Department of Internal Medicine, Division of Immunology/Allergy Section University of Cincinnati, Cincinnati, OH; 6 Department of Medicine, University of Washington, Spokane, WA; 7 Division of Allergy and Immunology, Department of Internal Medicine, University of Texas Southwestern Medical Center, Dallas, TX; 8 Division of Pulmonary and Critical Care Medicine and Allergy and Immunology, Department of Medicine, Medical University of South Carolina, Charleston, SC; 9 Department of Allergy and Immunology, University of Western Sydney and Campbelltown Hospital, Sydney, Australia; 10 Universitätsmedizin Berlin. Allergie-Centrum-Charité, Berlin, Germany; 11 Department of Allergy and Clinical Immunology, Ajou University School of Medicine, Suwon, South Korea; 12 Allergy Diagnostic & Clinical Research Unit, University of Cape Town Lung Institute, Groote Schuur, South Africa; 13 Division of Allergy and Clinical Immunology, Department of Medicine, Johns Hopkins University, Baltimore, MD; 14 Immunology Institute, Faculty of Medicine, Universidad Central de Venezuela, Caracas, Venezuela; 15 U.O. Allergologia e Immunologia Clinica, Fondazione IRCCS Ca' Granda, Ospedale Maggiore Policlinico, Milano, Italy

**Keywords:** urticaria, angioedema

## Abstract

Urticaria and angioedema are common clinical conditions representing a major concern for physicians and patients alike. The World Allergy Organization (WAO), recognizing the importance of these diseases, has contributed to previous guidelines for the diagnosis and management of urticaria. The Scientific and Clinical Issues Council of WAO proposed the development of this global Position Paper to further enhance the clinical management of these disorders through the participation of renowned experts from all WAO regions of the world. Sections on definition and classification, prevalence, etiology and pathogenesis, diagnosis, treatment, and prognosis are based on the best scientific evidence presently available. Additional sections devoted to urticaria and angioedema in children and pregnant women, quality of life and patient-reported outcomes, and physical urticarias have been incorporated into this document. It is expected that this article will supplement recent international guidelines with the contribution of an expert panel designated by the WAO, increasing awareness of the importance of urticaria and angioedema in medical practice and will become a useful source of information for optimum patient management worldwide.

## Ratification by Voting Member Societies of the World Allergy Organization October 2012

Albanian Society of Allergology and Clinical Immunology

American Academy of Allergy, Asthma and Immunology

American College of Allergy, Asthma and Immunology

Argentine Association of Allergy and Clinical Immunology

Argentine Society of Allergy and Immunopathology

Australasian Society of Clinical Immunology and Allergy

Austrian Society of Allergology and Immunology

Azerbaijan Society for Asthma, Allergy and Clinical Immunology

Brazilian Society of Allergy and Immunopathology

British Society for Allergy and Clinical Immunology

Bulgarian National Society of Allergology

Canadian Society of Allergy and Clinical Immunology

Colombian Allergy, Asthma, and Immunology Association

Croatian Society of Allergology and Clinical Immunology

Cuban Society of Allergology

Czech Society of Allergology and Clinical Immunology

Danish Society for Allergology

Dutch Society of Allergology

Egyptian Society of Allergy and Clinical Immunology

Egyptian Society of Pediatric Allergy and Immunology

Finnish Society of Allergology and Clinical Immunology

German Society for Allergology and Clinical Immunology

Honduran Society of Allergy and Clinical Immunology

Hong Kong Institute of Allergy

Hungarian Society of Allergology and Clinical Immunology

Icelandic Society of Allergy and Immunology

Indian College of Allergy, Asthma and Applied Immunology

Indonesian Society for Allergy and Immunology

Israel Association of Allergy and Clinical Immunology

Italian Society for Allergology and Clinical Immunology

Japanese Society of Allergology

Jordanian Society for Allergy and Clinical Immunology

Korean Academy of Allergy, Asthma and Clinical Immunology

Kuwait Society of Allergy and Clinical Immunology

Latvian Association of Allergists

Lebanese Society of Allergy and Immunology

Malaysian Society of Allergy and Immunology

Mexican College of Pediatricians Specialized in Allergy and Clinical Immunology Mongolian Society of Allergology

Norwegian Society of Allergology and Immunopathology

Panamanian Association of Allergology and Clinical Immunology.

Philippine Society of Allergy, Asthma and Immunology

Polish Society of Allergology

Romanian Society of Allergology and Clinical Immunology

Russian Association of Allergology and Clinical Immunology

(Singapore) Allergy and Clinical Immunology Society of Singapore

Slovenian Association for Allergology and Clinical Immunology

(South Africa) Allergy Society of South Africa

Spanish Society of Allergology and Clinical Immunology

(Sri Lanka) Allergy and Immunology Society of Sri Lanka

Swiss Society of Allergology and Immunology

(Thailand) Allergy, Asthma and Immunology Society of Thailand

Turkish National Society of Allergy and Clinical Immunology

Uruguayan Society of Allergology

Venezuelan Society of Allergy and Immunology

### Contributing Regional Member Societies

American Academy of Allergy, Asthma and Immunology

American College of Allergy, Asthma and Immunology

Asia Pacific Association of Allergy, Asthma and Clinical Immunology

European Academy of Allergy and Clinical Immunology

Latin American Society of Allergy and Immunology

## Introduction

Urticaria is a highly prevalent condition resulting in large numbers of medical consultations worldwide. Its prevalence ranges between 0.3 and 11.3% depending on the study population (see Prevalence section), and in recent years, an increase in the rate of hospitalizations due to urticaria and angioedema has been observed in some countries [1]. It has been estimated that approximately 20% of the population will experience an episode of acute urticaria (AU) at some point in their lifetime.

Although urticaria has a tremendous impact on patient's quality of life, it is often disregarded as a trivial disease by many physicians [2]. Therefore, patients are not adequately educated on the nature of their condition and its proper management, which involves not only pharmacological treatment but also the implementation of preventive measures to reduce the effects of various precipitating and aggravating factors.

This position paper provides updates on recent advances in the understanding of etiologic factors, pathogenic mechanisms, diagnostic methods, and medical management of acute and chronic urticaria (CU) and angioedema.

## World allergy organization global position papers

The World Allergy Organization (WAO) is an international federation of 89 regional and national allergy and clinical immunology societies dedicated to raising awareness and advancing excellence in clinical care, research, education, and training in allergy and clinical immunology. This WAO position paper on the diagnosis and treatment of urticaria and angioedema was developed as a document presenting a worldwide perspective encompassing the participation and input of leaders from all WAO regional member societies.

This position paper includes sections on the definition, prevalence, classification, mechanisms, diagnosis, treatment, and prognosis of urticaria and angioedema. In addition, special chapters dealing with particularly important issues have been included to review physical urticarias, urticaria in childhood, urticaria and pregnancy, and quality of life and patient-reported outcomes (PROs). The concept of disease control for CU, similar to other chronic allergic diseases such as asthma and rhinitis, is highlighted and the importance of patient education on the possible mechanisms, causes, prognosis, and treatment of acute and CU is emphasized.

National and regional guidelines for the diagnosis and treatment of urticaria and angioedema have been previously published [3-5]. Because urticaria and angioedema are a frequent cause for consultation not only in allergology clinics but also in general practitioners' offices, and these diseases are often underestimated by physicians, it was important to provide useful orientations for the management of these vexing conditions.

The objectives of this WAO position paper on urticaria and angioedema are to provide updated information on the assessment and treatment that should be applied in health care settings worldwide to obtain a better symptom control, improve patients' quality of life, contribute to patient education, and enhance accessibility to more effective therapies. This information is designed for use by both allergy and immunology specialists as well as physicians in general practices who daily observe patients with urticaria and angioedema.

## Methods

This position paper was developed by a special steering committee of internationally recognized experts appointed by the WAO Scientific and Clinical Issues Council.

Recommendations are based on the best evidence presently available. Urticaria and angioedema guidelines previously published in indexed peer-reviewed journals were reviewed. Drafts were developed through e-mail correspondence among authors, distributed to all members of WAO Board of Directors for comment, and then circulated to WAO Member Societies for review, comments, and approval. In all, more than 50 allergy and immunology specialists on 5 continents contributed to the development of this position paper.

## Definition and Classification

Urticaria is characterized by the sudden appearance of wheals and/or angioedema, defining wheals as a cutaneous swelling of variable size, almost invariably surrounded by a reflex erythema, with associated itching or, sometimes, a burning sensation, and of transient nature, with the skin returning to its normal appearance in usually 1 to 24 hours.

Angioedema can be defined as a sudden and pronounced swelling of the deep dermis and subcutaneous tissue or mucous membranes, with a painful rather than an itching sensation and a slower resolution than for wheals that can take up to 72 hours [4,6].

### Classification

Urticaria can be classified on the basis of its duration and in the presence or absence of inducing factors (induced vs spontaneous).

#### Duration

AU is characterized by the occurrence of hives and/or angioedema for < 6 weeks, whereas episodes lasting longer than 6 weeks are regarded as CU [7]. This somewhat arbitrary distinction of 6 weeks becomes important in regard to potential mechanisms, approaches to evaluation, and options for treatment. The classification of urticaria is presented in Table 1.

**Table 1:** Classification of Urticaria Subtypes (Presenting With Wheals and/or Angioedema) Based on the Different Eliciting Stimuli

Types	Subtypes	Definition
Spontaneous urticaria	Acute spontaneous urticaria	Spontaneous wheals and/or angioedema < 6 wk
	Chronic spontaneous urticaria	Spontaneous wheals and/or angioedema > 6 wk
Urticarias induced by physical agents	Cold contact urticaria	Eliciting factor: cold objects/air/fluids/wind
	Delayed pressure urticaria	Eliciting factor: vertical pressure (wheals arising with a 3-12 h latency)
	Heat contact urticaria	Eliciting factor: localized heat
	Solar urticaria	Eliciting factor: UV and/or visible light
	Urticaria factitia/dermographic urticaria	Eliciting factor: mechanical shearing forces (wheals arising after 1-5 min)
	Vibratory urticaria/angioedema	Eliciting factor: vibratory forces, e.g. pneumatic hammer
Other inducible urticarias	Aquagenic urticaria	Eliciting factor: water
	Cholinergic urticaria	Elicitation by increase of body core temperature due to physical exercises, spicy food
	Contact urticaria	Elicitation by contact with urticariogenic substance
	Exercise-induced anaphylaxis/urticaria	Eliciting factor: physical exercise

Modified with permission from Zuberbier et al [4]. Copyright 2009 John Wiley & Sons.

Urticaria pigmentosa (cutaneous mastocytosis), urticarial vasculitis, familial cold urticaria, and nonhistaminergic angioedema (eg, hereditary or acquired C1 esterase inhibitor deficiency) are no longer considered as subtypes of urticaria, due to their distinctly different pathomechanisms [4].

Finally, there are syndromes that can be associated with wheals:

• Muckle-Wells syndrome: a combination of wheals, deafness, and amyloidosis, characterized by sensorineural deafness, recurrent urticaria, fever, and arthritis [8].

• Schnitzler syndrome: chronic wheals and monoclonal gammopathy (usually IgM) associated with at least 2 of the following components: fever, arthralgia, or arthritis, bone pain, hepatomegaly or splenomegaly or hepatospleenomegaly, lymphadenopathy, elevated erythrocyte sedimentation rate, leukocytosis, and/or abnormal findings on bone morphological investigations [9].

• Gleich syndrome: episodic angioedema with eosinophilia [10].

• Well syndrome or eosinophilic cellulitis: granulomatous dermatitis with eosinophilia [11].

## Prevalence

The prevalence of urticaria and angioedema varies according to the population under investigation. Lifetime prevalence rates of 8.8% have been reported, with a 1.8% rate for CU [12]. Approximately 10 to 20% of the population will experience an episode of AU at some point in their lifetime, and 0.1% will develop chronic spontaneous urticaria [13].

In a study carried out in Spain, the prevalence of urticaria in the past 12 months was 0.8%, and the prevalence of CU was 0.6%. Urticaria was present more often in female patients among the 35 to 60 years age-group (mean age, 40 years). Duration of the disease was 1 to 5 years in 8.7% of the patients and more than 5 years in 11.3% [14].

Autoimmune disturbances are present in 40 to 45% of patients with chronic spontaneous urticaria [15]. Angioedema is present in 40 to 50% of cases of chronic spontaneous urticaria, 10% of patients experience only angioedema without hives and 40% exhibit wheals alone [6,13,16]. Recently, an increase in the rate of hospital admissions for angioedema (3.0% per year), and urticaria (5.7% per year) has been observed in Australia. Admissions for urticaria were 3 times higher in children aged 0 to 4 years. The greatest increase in hospitalizations for urticaria was present in those aged 5 to 34 years (7.8% per year), and for angioedema, it was higher in patients 65 years and older [1]. It is not known if this increase has occurred in other countries.

## Etiology and Pathogenesis

Symptoms of chronic spontaneous urticaria appear seemingly spontaneously, that is, in most patients, there is no identifiable exogenous stimulus for hive production. In some patients, however, nonspecific exogenous triggers for the development of wheals and/or angioedema, such as exercise, environmental changes, and stress are present. We now consider this group to be chronic "spontaneous" urticaria [4,17]; thus, if an etiology is to be found, it is likely to be endogenous, leading to the resultant cutaneous inflammation that is expressed as a hive.

### Psychosomatic Factors

For decades, theories regarding etiology would appear and disappear, but none proved to be credible. In the 1950s and 1960s, many considered chronic spontaneous urticaria to be an anxiety disorder, and even now, there is some limited data to suggest worsening of symptoms with anxiety. It is now generally accepted that mental illnesses, such as depression and anxiety, influence the quality of life of chronic spontaneous urticaria patients and those are important comorbidities in such patients. However, it cannot be considered to be a cause, and a clear distinction between less tolerance of symptoms and actual worsening of skin inflammation has not been made.

### Type 1 Food Allergy

The relation between food allergy/pseudoallergy and CU is controversial because some experts do not recommend elimination diets for such condition, whereas others have observed the improvement of symptoms by means of pseudoallergen-free diets in about one third of patients with chronic spontaneous urticaria [18].

### Autoreactivity and Autoimmunity

Autoreactivity (see below) represents one major approach to elucidating the initiating stimulus for persisting hive formation. It is clear that cutaneous mast cell degranulation induces hive formation and on biopsy, a nonnecrotizing perivascular infiltration of cells is seen, which resembles a cutaneous late phase reaction [19-21]. There is infiltration with granulocytes (neutrophils, eosinophils, and basophils), although the magnitude can vary considerably. T cells are very prominent; most are CD4^+ ^with a mixture of TH1 and TH2 subtypes [21]. There are also monocytes, but very few, if any B lymphocytes. A similar infiltration of cells can be seen when serum of patients is injected intradermally into their own skin, with a resultant wheal and flare reaction termed autoreactivity [22]. This is seen in 30% of patients and led to considerations of autoimmune (ie, immunoglobulin) mechanisms for the initiation of mast cell degranulation. At first, 5 to 10% of patients were found to have circulating IgG anti-IgE, which is functional,[23] and subsequently, 30 to 40% of patients were found to have IgG antibody to the *α *subunit of the IgE receptor [24]. The thesis is that cross-linking IgE receptors or occasionally IgE itself could activate skin mast cells in a selective fashion. Most commonly, human basophils were employed as an alternative to cutaneous mast cells and worked well to identify what has been termed chronic autoimmune urticaria. Serum-evoked basophil histamine release (HR), although time consuming, is the most quantitative assay, but upregulation of CD63 or CD203 assessed by fluorescence-activated cell sorter analysis can also be employed.

The remaining 55 to 60% of patients lacking such autoimmunity are considered to have chronic nonautoimmune or idiopathic (but nevertheless spontaneous) urticaria. In vitro studies support antireceptor antibody binding to the a subunit of the IgE receptor to activate the classical complement pathway with release of C5a, which further activates basophils and mast cells and contributes to recruitment of granulocytes and monocytes by its chemotactic activity [25]. Marked reduction in serum complement levels or complement deposition in lesion biopsies have not been demonstrated in subjects with serum autoimmunity.

The presence of these antibodies does not prove causality, although their role as a pathogenic mechanism is debated with evidence pro and con [26,27]. Clearly, more than half of the patient population with chronic spontaneous urticaria lacks these anti-FceRI autoantibodies. However, in vitro HR can be blocked completely by saturating IgE receptors with an IgE myeloma protein so that antireceptor antibodies are sterically prevented from binding,[28] although an occasional exception is noted [29]. Soluble *α *subunit can be added to serum to bind the antireceptor antibody so that HR is prevented [24,30]. In most cases studied, isolation of IgG has reproduced basophil activation based on HR, although the IgG depleted serum is negative.

There are also publications suggesting the presence of vasoactive factors in IgG-depleted serum of patients with CU,[31] but no factor has been isolated or identified, and the assay employed for detection is more typically the autologous skin test rather than basophil HR. Plasmapheresis can be used to stop the urticaria acutely indicating that removal of a critical plasma factor can potentially stop symptoms in select cases [32].

### Possible Role of Immunoglobulin E

Finally, it was theorized that anti-IgE therapy with omalizumab might be effective in patients with hives. The thesis was that as IgE levels decrease toward zero, IgE receptors are downregulated, and if the spacing and surface density is sufficiently low, the IgG anti-*α *subunit cannot cross-link receptors and activation of basophil and mast cell would not occur. In practice, the IgE receptor reduction via omalizumab occurs rapidly for blood basophils and much more slowly on skin mast cells, yet omalizumab does not eliminate either cell's capacity to respond to a cross-linking stimulus [33-35]. Thus far, therapy with this monoclonal antibody has been extremely successful,[36-38] and phase 3 studies of its efficacy and safety are ongoing currently. The mechanism of its effect is not clear because some patients respond dramatically in 2 to 3 days; so fast that receptors could not be significantly down-regulated, and there is evidence of efficacy even in the non-autoimmune urticaria population [39,40]. Thus, it is likely that some unknown role for IgE is operative in all these patients, whereas receptor downregulation is superimposed some weeks later. There is precedent for synthesis of IgE that is either intrinsically abnormal or perhaps reactive with an unknown autoantigen; for example, it has been shown that isolated monomeric IgE of some patients with cold urticaria can passively transfer the disease,[41] that is, the IgE binds to normal mast cells of a recipient and renders them "cold sensitive" so that mast cells then degranulate upon a change in temperature. The abnormality resides with the IgE not the mast cell. There is also evidence for heightened skin mast cell release in active CU subjects [42-44]; furthermore, a recent publication reports the presence of a nonimmunoglobulin factor in patient's sera capable of activating cultured mast cells in vitro [45].

### Additional Observations on the Pathogenesis of Urticaria

There are additional observations regarding chronic spontaneous urticaria possibly related to pathogenesis. Early on, an association with Hashimoto thyroiditis, and more specifically, with the presence of autoantibodies was reported,[46,47] including antiperoxidase and antithyroglobulin antibodies. Although IgE antithyroid antibodies could have pathogenic significance, most patients have only IgG antibodies, and their presence is thought to represent a proclivity to autoimmune phenomena and a possible marker for the presence of anti-IgE receptor antibody [48]. C-reactive protein is elevated in the group when compared with normals, suggesting systemic recognition of cutaneous inflammation. Matrix metalloproteinase levels are increased in the blood plasma perhaps originating from skin inflammation [49]. The extrinsic coagulation cascade is activated based on elevated prothrombin fragments 1 and 2 and D-dimer levels but without any abnormal coagulopathy [50,51]. Tissue factor, although produced by activated endothelial cells (stimulated, for example, by histamine or leukotriene C4), nevertheless seems to be secreted by eosinophils within the tissue [52]. It has been theorized that thrombin might activate mast cells; however, active thrombin has not been found; most demonstrations of thrombin-induced HR have employed rodent mast cells, and it is not clear that the thrombin dose needed is physiologic [53,54]. Leukotriene C4, cytokines, and growth factors have also been found to be elevated in plasma of patients with CU, and cellular adhesion molecules are upregulated [55-57]. It is not clear whether these inflammatory stigmata are produced by activated cells in blood or these are found to having been produced in the skin.

Another approach to understanding chronic spontaneous urticaria, whether associated with autoantibodies, is to focus on possible abnormalities within the cell, and the basophil is a prime candidate. A hallmark of CU is the unique relationship of disease activity to altered blood basophil phenotypes [58]. Since the 1970s, many groups have found that blood basophils from active chronic spontaneous urticarial (CSU) subjects have reduced IgE receptor-mediated HR but not in the HR induced via IgE receptor-independent pathways (ionophore, 48/80, N-formyl-methionyl-leucin-phenylalanine (fMLP), bradykinin and monocyte chemoattractant protein 1 (MCP-1), indicating a specific defect in the Fc*ε*RI pathway [59-62]. CSU subjects can be segregated based on the bimodal distribution of their basophil FcεRI-induced HR profiles, a feature that is stable during active disease [60,63]. Fifty percent of CSU subjects have significant reductions in their blood basophil IgE receptor-induced HR (< 10% of total histamine content) and are defined as nonresponders. The remaining CSU subjects have > 10% basophil Fc*ε*RI-induced HR and are called responders [64]. These basophil subgroups also have altered protein levels of signaling molecule expression that reflects their IgE receptor functional classification. Blood basopenia is also unique to CSU and is correlated with disease activity [65,66]. Furthermore, basophils are found in both lesional and nonlesional skin biopsies of CSU subjects, suggesting that basopenia is related to the recruitment of basophils to skin tissues [67]. Systemic corticosteroid therapy, which leads to an increase in blood basophil numbers and reduces skin symptoms in CSU, is known to inhibit basophil recruitment to the skin [66,68,69]. In CSU subjects who enter remission, basophils shift toward normalization of basophil IgE receptor-mediated HR and correction of basopenia [60,63].

## Diagnostic Approach to Urticaria

The goal of diagnostic measures is to (1) identify urticaria type and subtype and (2) identify underlying causes (in long-standing or severe chronic sponganeous urticaria only). Urticaria of either acute or chronic type is a common disease that manifests with heterogeneous phenotypes. It poses a high socioeconomic burden for patients [70]. In general, a limited initial workup is indicated, unless the clinical history dictates otherwise.

AU is more common than the chronic form and is associated with a rapid recovery, but the identification of its etiology can be helpful to prevent recurrence especially when allergy is suspected to be the cause. Although chronic spontaneous urticaria has various etiologies and subtypes, routine patient evaluation comprising the careful acquisition of patient history, physical examination, and ruling out of systemic diseases should be considered. Specific provocation and laboratory tests are needed to confirm the underlying causes whenever the clinical history is supportive. These extensive diagnostic procedures should be considered on an individual basis in patients with long-standing, severe, or persistent urticaria.

### Diagnostic Approach for Patients With Acute Spontaneous Urticaria

Although both a detailed history and physical examination remain essential, the etiology of acute spontaneous urticaria can be suggested by a patient's history. Upper respiratory tract and viral infections are the most common etiology in children. Foods and drugs such as antibiotics and nonsteroidal anti-inflammatory drugs (NSAIDs)[71] can be considered for both adults and children. In general, diagnostic workup is indicated only when type I allergy is suspected to be the underlying cause of acute spontaneous urticaria.

### Diagnostic Approach in Patients With CSU

In patients with CSU, it is necessary to obtain a thorough history, including all possible eliciting factors, and to identify the significant aspects of the disease. This initial assessment is helpful in the identification of the subtype of urticaria (acute vs chronic, spontaneous vs induced). The overall duration of CSU is likely to be longer in patients with high disease severity, angioedema, positive autologous serum skin test (ASST) results, or comorbidity with physical urticaria. Next, the impact of the disease on the patient and the disease activity should be evaluated using the urticaria activity score and the CU quality of life questionnaire (see Quality of Life and Patient-Reported Outcomes). The patient should be asked about the time of onset; frequency and duration of wheals; presence of diurnal variation; shape, size, and distribution of wheals; associated angioedema; family and personal history of urticaria; atopy; medications (NSAIDs, hormones, laxatives, immunizations); and observed correlation with food and stress. The first step is to exclude major comorbid disorders and physical urticaria, and the second step is to identify the underlying cause. Patient questioning together with physical examination and laboratory and provocation tests may be useful to identify associated diseases and comorbidities, and in some cases, the underlying cause. Routine laboratory testing in the absence of a clinical history is rarely helpful in determining an etiology for patients with CSU [72,73]. Nevertheless, expert opinion differs in regards to the number and type of testing appropriate for patients with CSU.

Routine hematological tests, including complete blood count and liver function tests, the determination of erythrocyte sedimentation rate, and C-reactive protein levels may be considered. The role for infectious agents such as *Helicobacter pylori *in the causation of chronic urticarial is controversial, and the evidence is weak and conflicting [74]. Screening for thyroid autoimmunity may be considered. Although type I allergies are a very rare cause of CSU, the IgE-mediated mechanism may be considered in patients with intermittent symptoms. For differential diagnosis from patients with angioedema alone without wheals, the measurement of C4 and C1 esterase inhibitor levels may be necessary. About one third of CSU patients have aspirin/NSAID hypersensitivity, and oral provocation tests with aspirin are available to confirm this if needed [71]. Some CSU patients improve with a food-additive-free diet, and challenge tests with food additives may be necessary [18]. The ASST is the only generally available test to screen for autoantibodies against either IgE or the high-affinity IgE receptor. Autoimmune urticaria responds poorly to H1 antihistamines and often manifests as severe CSU. However, some studies have demonstrated low sensitivity of the ASST with a high false-positive rate. The basophil HR test is more refined but is also insufficiently sensitive to be applied routinely. The diagnostic workup should include physical stimulation tests if physical urticaria is suspected. Ice cube or cold water tests are used widely for cold urticaria, and exercise challenge tests are used for cholinergic and exercise-induced urticaria. To improve outcomes for CSU patients, quality of life and psychiatric comorbidity should be considered. A skin biopsy may be needed to confirm urticarial vasculitis and Schnitzler syndrome.

## Treatment

### Antihistamines

#### Second-Generation Antihistamines at Licensed Doses

Second-generation antihistamines (azelastine, bilastine, cetirizine, desloratadine, ebastine, fexofenadine, levocetirizine, loratadine, mizolastine, and rupatadine) at licensed doses represent the mainstay of treatment for urticaria. A number of high-quality, randomized, controlled trials have been carried out with these drugs in patients with mild/moderate urticaria [3,5,75]. Evidence of their effectiveness is very high. They are also safe and well-tolerated.

#### Comparative Efficacy of Second-Generation Antihistamines

The higher efficacy of cetirizine (10 mg) over fexofenadine (180 mg) has been shown in a randomized, double-blind study [76]. In another multicenter, randomized, double-blind study, levocetirizine was more effective than desloratadine [77]. Bilastine and levocetirizine have been recently compared in a randomized double-blind study and showed a similar effectiveness [78].

Finally, in a series of in vivo comparative studies assessing suppression of histamine-induced wheal and flare responses of different second-generation antihistamines, cetirizine and its derivative levocetirizine were always superior to other nonsedating antihistamines in terms of efficacy [79-81]. However, a new study did not demonstrate significant differences between overall inhibition of wheal or flare by 20 mg of bilastine and 10 mg of cetirizine [82]. The correlation of these in vivo comparisons with clinical efficacy is unknown. Randomized, double-blind, placebo-controlled trials have not found relevant differences in sedation and impaired psychomotor function between levocetirizine, cetirizine, and loratadine [83]. Some clinical trials and postmarketing surveillance studies found that the sedative effect of cetirizine was greater than that of fexofenadine or loratadine [84].

#### First-Generation Antihistamines

Double-blind placebo-controlled studies have demonstrated efficacy for several first-generation antihistamines in CU with overall similar efficacy to second-generation antihistamines [85-87]. First-generation antihistamines have been recommended as add-on therapy to CU patients who have had inadequate control on second-generation antihistamines; however, studies to demonstrate efficacy of this approach are lacking [28]. Sedation and cognitive/psychomotor function impairment are side effects of first-generation antihistamines, but the degree of these side effects varies between individuals [88]. Therefore, sedating antihistamines are typically recommended to be dosed as a single nocturnal dose to reduce daytime impairment [89]. Studies have shown that tolerance to performance impairment improves while taking first-generation antihistamines after 3 to 5 days of treatment [85,90]. Based on the availability, cost-effectiveness, and safety of second-generation antihistamines, first-generation antihistamines are being now less frequently recommended as first-line agents [3,4,75,91,92]. In other words, first-generation antihistamines do not provide additional benefits to those obtained with nonsedating antihistamines.

#### Dosing of Second-Generation Antihistamines at Higher Than Licensed Recommendations

Many patients with CU may not respond adequately to the recommended doses of second-generation antihistamines. Limited data are available on dosing second-generation antihistamines at higher than the recommended amounts [93-96]. An open-label study of cetirizine [93] and a double-blind, controlled study of desloratadine in patients with cold urticaria [96] demonstrated that increased dosages of these second-generation antihistamines had greater therapeutic benefits without increased side effects. Subsequently, a double-blind multicenter study in CU patients using desloratadine and levocetirizine was published showing improved effectiveness with higher dosing up to 4 times the recommended amount [97]. Although a double-blind placebo-controlled study did not show differences in efficacy between a 10 mg or 20 mg daily dose of rupatadine in CU,[98] a recent study showed that higher doses of this drug are more effective than standard doses [99]. Similar studies have not been performed or verified with other second-generation antihistamines. In patients with CSU, updosing of nonsedating antihistamines increases the rate of response from about 45% to more than 60%. Due to their good tolerability and safety present, recommendation for patients who do not respond to standard doses of nonsedating antihistamines is to use higher doses instead of corticosteroids as second-line treatment.

#### H2-Antagonists

Most studies demonstrating efficacy of H2-antagonists added to H1-antagonists in CU have been performed with cimetidine [100-102]. Studies evaluating the combination of H1-antagonists and ranitidine in CU have yielded conflicting results [103,104]. Cimetidine's effectiveness is believed to be due to its ability to inhibit a number of cytochrome p450 isoenzymes involved with the metabolism of first-generation antihistamines, resulting in an increased plasma concentrations of antihistamines like hydroxyzine [105,106]. These additive effects have not been seen with the combination of cimetidine and cetirizine, and studies evaluating the combination of H1-antagonists and ranitidine or famotidine have yielded conflicting results [103,104]. Thus, altogether the quality of evidence for the use of H2 receptor antagonists in association with H1 antihistamines is low, and such association does not seem to produce any advantage over the use of anti-H1 antihistamines alone; however, other experts consider the combination to be safe and affordable, sometimes effective, and preferable in its risk-benefit profile to other second-line treatment options [106].

### Leukotriene Receptor Antagonists

The effectiveness of these drugs has been reported in several relatively small, randomized, double-blind studies,[108-112] but the results have been inconsistent [113]. A recent review on this issue concluded that leukotriene receptor antagonists might be effective in subsets of patients with CSU associated with aspirin or food additive intolerance or positive on ASST but not in other patients with chronic spontaneous urticaria,[114] although other studies do not seem to support this view [115]. Altogether, existing evidence of their effectiveness is limited, and the grade of recommendation for their use is low. Nonetheless, these drugs may be tried in patients unresponsive to antihistamines in view of their excellent safety profile.

### Corticosteroids

Although it is clinically recognized that oral corticosteroids are effective for H1-antihistamine-resistant CU, controlled studies are lacking [117]. In view of the potentially severe side effects associated with long-term treatment, oral corticosteroids should be used for short periods and at the minimally effective dose necessary to achieve control. There is no consensus on the dose and duration of oral corticosteroids for the management of CU, but some recommended approaches about short-term therapy have been published [117]. Attempts should be made to find alternative agents to control urticaria to avoid long-term corticosteroid use. In rare patients, long-term corticosteroid use may be justified; however, patients should be monitored closely for adverse effects of corticosteroid therapy.

One published protocol suggests using prednisone 15 mg daily (preferably 10 mg) and decrease by 1 mg (using 1-mg tablets) each week. Considerable efficacy can be achieved, and subsequent responsiveness to other modalities can be enhanced. If higher doses are needed to significantly lessen symptoms, the drug should not be used [15,117]. In conclusion, corticosteroids should be used sparingly only when all other therapies failed, until other controller therapies can be found that control the hives.

### Anti-Inflammatory Agents

Although the evidence for efficacy in the treatment of CU for many of the following anti-inflammatory agents is limited, the favorable cost and relatively safe side effect profiles warrant their consideration before using more expensive or more toxic agents.

#### Dapsone

Case reports and case series have found dapsone to be effective in the treatment of CU, idiopathic angioedema, delayed pressure urticaria, and urticarial vasculitis [118-124]. A recent randomized, unblinded study of 65 CU patients compared dapsone and desloratadine with desloratadine alone over a 3-month treatment period followed by a 3-month post-treatment observational period [125]. The dapsone-treated group had similar reductions in urticaria scores compared with the desloratadine monotherapy group, but 9 dapsone-treated patients experienced complete responses, whereas none of the control subjects did. Five of 9 responders remained urticaria free 3 months after discontinuing dapsone. Dapsone is usually well-tolerated but has predictable side effects including dose-related anemia. Less common adverse effects include peripheral neuropathy, rash, gastrointestinal complaints, hepatotoxicity, and rarely methemoglobinemia, blood dyscrasias, or the syndrome of drug rash with eosinophilia and systemic symptoms [126]. Before the initiation of dapsone therapy, glucose-6-phosphate dehydrogenase levels should be normal as the risk of severe hemolysis is increased in glucose-6-phosphate dehydrogenase-deficient patients. Laboratory monitoring for anemia and hepatotoxicity is recommended for patients on dapsone [127].

#### Sulfasalazine

Case reports and case series have suggested that sulfasalazine is efficacious in patients with CU and delayed pressure urticaria [128-130]. A retrospective observational study of 19 CIU patients demonstrated significant improvement in 14 of 19 patients with more modest benefit in 4 additional patients [131]. Therapeutic response occurred within 1 month, and doses above 2 g/day had no additional benefit. As stated, most references to sulfasalazine use in CU are case reports or uncontrolled studies.

The most common side effects include nausea, vomiting, dyspepsia, anorexia, and headache [132]. These symptoms typically occur early in therapy and are more common in patients taking > 4 g/day, which is beyond the dose recommended for the treatment of CU. Slow-dosing escalation regimens over several days may reduce the gastrointestinal effects. Hematologic abnormalities, proteinuria, and hepatotoxicity are uncommon, but laboratory monitoring for these adverse effects is recommended [133].

#### Hydroxychloroquine

Limited data are available on the use of hydroxychloroquine in CU. A case report suggested efficacy in a patient with hypocomplementemic urticarial vasculitis [134]. A randomized, blinded, placebo-controlled study of 21 CU subjects demonstrated significant improvement in the quality of life but only trends toward improvement in urticaria activity scores or reduction in other medications [135]. Hydroxychloroquine is generally well-tolerated with the most worrisome adverse effect being retinopathy. The risk of retinopathy from hydroxychloroquine is exceedingly rare [136]. Almost all cases have occurred in high-risk individuals who have used the drug > 5 years. The most recent American Academy of Ophthalmology [137] guidelines recommend that all patients have a baseline ophthalmologic examination within the first year of starting the drug and annual screening after 5 years or a cumulative dose of > 1000 g. For higher risk patients including the elderly and patients with kidney/liver dysfunction, retinal disease, or maculopathy, annual eye examinations are recommended.

#### Colchicine

CU patients with neutrophilic inflammation responded to colchicine,[138] and case reports suggest its efficacy in patients with urticarial vasculitis [139-141]. Colchicine is generally well-tolerated with the most frequent adverse effect being diarrhea. High doses can cause bone marrow suppression, and long-term use can rarely cause myopathy and neuropathy.

### Immunosuppressive Agents

#### Calcineurin Inhibitors

Case reports and case series have described benefit of cyclosporine to patients with CU unresponsive to antihistamines [142-144]. There are 4 published randomized, double-blinded, controlled trials investigating the therapeutic utility of cyclosporine for patients with CU/angioedema who had failed second-generation antihistamines [145-148]. Although the results of these studies show favorable effects, the side effects of this agent may outweigh its benefits. Further research is necessary to determine the effect of cyclosporine in the treatment of more well-defined refractory CU patients. The optimal dose of cyclosporine has not been adequately delineated. Investigators have initiated therapy using both higher doses (eg, 3-5 mg/kg per day) versus lower doses (200 mg/day). During the treatment period, blood pressure, kidney function, and liver function should be regularly monitored. In a follow-up study after stopping cyclosporine, complete remission lasted up to 9 months in about 50% of patients and a decreased number of flare-ups and a restored response to antihistamine treatment was observed in some subjects [148]. Recently, a low-dose, long-term maintenance therapy for up to 2 years has been suggested for those who show a marked propensity to relapse after discontinuation [149].

Tacrolimus, another calcineurin inhibitor, has been reported in an observational study to be effective in CU patients unresponsive to antihistamines, one of which was also unresponsive to cyclosporine [150].

#### Other Immunosuppresive Agents

Several other immunosuppressive drugs, including methotrexate, cyclophosphamide, azathioprine, sirolimus, and mycophenolate mofetil, have been used to treat H1-antihistamine-resistant CU. However, most experience relies on case reports or single-center uncontrolled studies. Two recent retrospective studies have been published showing that methotrexate at a weekly mean dosage of 15 mg is effective and safe in the majority of CU patients who are not responsive to conventional therapy [151,152]. According to Perez et al,[151] methotrexate exerts anti-inflammatory and immunosuppressive effects and may therefore benefit CU independently of the pathogenic mechanism, whether associated with autoantibodies. The efficacy of intravenous and oral cyclophosphamide [153,154] and azathioprine [155] has been demonstrated in case reports who had antihistamine-resistant CU and were positive on ASST. Both drugs have been successfully employed in the treatment of urticarial vasculitis [156]. Mycophenolate mofetil seems to be a useful treatment option for patients with CU who do not respond to antihistamines and/or corticosteroids with experience limited to observational studies [157,158].

### Biological Agents

#### Omalizumab

Recently, a growing number of studies evaluating the effectiveness of omalizumab (humanized monoclonal anti-IgE antibodies) in different subsets of antihistamine unresponsive CU/angioedema patients have been reported [36,39,159-166]. Although the current experience with omalizumab in the treatment of CU is encouraging, rare cases of omalizumab failure have been reported [167]. Several multicenter, randomized, placebo-controlled, dosing studies are still in progress to assess the role of this agent, but some have very recently appeared in the literature [38,40]. Efficacy and side effect profile potentially make omalizumab the future drug of choice for refractory chronic spontaneous urticaria. The main limitations of omalizumab treatment include limited availability, high-cost, and long-term clinical benefits.

#### Intravenous Immunoglobulin

Success in CU was first reported in an open-label trial of 10 CU patients with positive ASST and basophil HR tests who failed other therapies at a dose of 0.4 g/kg per day for 5 consecutive days [168]; 9 of 10 patients improved with 3 patients experiencing prolonged remission after a 3-year follow-up. Other case reports and case series have found intravenous immunoglobulin (IVIG) to be effective,[169,170] whereas others have not [171,172]. IVIG can be dosed in several ways, but the optimal dose, number of infusions, and frequency are not fully delineated. One study using low-dose IVIG (0.15 g/kg every 4 weeks) resulted in an improvement in 26 of 29 patients including 19 who experienced complete remission [173]. IVIG may be effective for delayed pressure urticaria and angioedema,[174] solar urticaria,[175] and urticarial vasculitis [176]. IVIG is relatively safe with predictable infusion-related adverse reactions including headache, myalgias, and nausea and rarely anaphylaxis, aseptic meningitis, or renal failure. In general, IVIG should be reserved for patients refractory to other alternative therapies.

### Other Therapies

Anticoagulants have recently been found to be effective in patients with refractory CU [50-52,177-180]. One recent study reported that low-molecular weight heparin was effective in a subset of refractory CU patients with elevated D-dimer levels [181]. Despite this increasing evidence, anticoagulant therapy cannot be presently recommended as a routine treatment for CU.

Other therapies have been reported as cases or case series for the treatment of CU, and very little information is known about their effectiveness and therefore is not recommended for routine use. These treatments include theophylline, androgens, β-agonists, nonsteroidal antinflammatory drugs, tumor necrosis factor-*α *inhibitors, calcium channel blockers, gold, plasmapheresis,[32] phototherapy, and autohemotherapy.

## Note about the quality of evidence and strength of recommendation grading

All existing systems to grade the quality of evidence and the strength of recommendations have their own weaknesses. In the present article, the GRADE system [182] has been adopted. One of the problems of this system is that in its original form, it has only 2 strengths of recommendation, that is, weak or strong. Thus, to indicate which "weak recommendations" are stronger than others (ie, have a recommendation in between weak and strong or in other words "moderate") the special notation "**" has been adopted (see Table 2).

**Table 2:** Quality of Evidence and Strength of Recommendation for Use of Intervention Based on the GRADE System [182] (Updated to August, 2011)

Drug	Quality of Evidence	Strength of Recommendation
Second-generation antihistamines (at licensed doses)	High	Strong (+)
First-generation antihistamines	High	Strong (-)
Second-generation antihistamines (at higher than licensed doses)	Moderate	Weak (+)
Anti-H2-antihistamines as add-on therapy	Moderate	Weak (+)
Oral corticosteroids (short course)	Low	Weak (+)
Oral corticosteroids	Very low	Strong (-)
Leukotriene receptor antagonists (as add-on therapy)	Low	Weak (+)
Anti-inflammatory agents (dapsone, sulfasalazine, hydroxychloroquine, colchicines, mycophenolate mofetil)	Low-very low	Weak (+)
Immunosuppressive agents		
Cyclosporine	Moderate	Weak (+)*
Methotrexate	Very low	Weak (+)
Cyclophosphamide	Very low	Weak (+)
Biologic agents		
Omalizumab,	Moderate	Weak (+)*
IVIG	Low	Weak (+)

(+), recommendation for medication; (-), recommendation against medication.

*Although the recommendation is "weak" according to the GRADE approach, it is stronger than in other cases based on the quality of existing evidence.

## The prognosis of urticaria and angioedema

The prognosis of AU is excellent, with most cases resolving within days; however, the prognosis of CU is variable. If angioedema is present, the prognosis is worsened (see below). CU is more common in adults and unusual in children.

### Acute urticaria

Few studies are available on the prognosis of AU [183,184]. Two studies indicated that 20 to 30% of young children with AU are at the risk of chronic or recurrent urticaria [185-187]. More concerning than repeated episodes of AU is the progression of the disease to CU [188,189].

Hospital admissions for urticaria were approximately 3 times higher in children aged 0 to 4 years than for other ages. Between 1993-1994 and 2004-2005, there were significant increases in the rate of hospital admissions for urticaria in all ages [1].

In adults, longer disease duration is an important risk for poorer prognosis [190]. AU causes discomfort, but not mortality, unless associated with angioedema of the upper airways [191-193]. Morbidity depends on severity and duration. One study found urticaria patients can have as much psychological, social, and occupational distress as patients awaiting triple coronary artery bypass surgery [194]. If a patient continues to be exposed to a trigger, urticaria may become chronic.

### Chronic Urticaria

Studies in multiple countries report complete resolution in approximately one third of patients with idiopathic CU for more than 1 to 5 years and partial improvement in another third [195].

Spontaneous remission occurs in 30 to 50% of patients within 1 year, and another 20% within 5 years. Nearly 20% of patients still have symptoms after 5 years. Almost half of patients with CU lasting 6 months are likely to have wheals 10 years later [196]. Those with more severe symptoms may have longer lasting disease. A retrospective study of 372 patients with severe urticaria described resolution of symptoms in 29% of patients after 5 years and 44% after 10 years [197,198].

Patients younger than 30 years with more severe symptoms, or symptoms with physical causes, fared less well [195]. For those with physical urticarias, their condition may be better measured in decades, rather than years, but can typically be controlled [199].

In an Amsterdam prospective study of 220 patients with CU and angioedema,[200] 35% of patients had complete resolution of symptoms 1 year after enrollment. Resolution rates ranged from a high of 59.6% in patients with idiopathic urticaria-angioedema to a low of 16.4% in patients who had urticaria with a physical cause [195]. In a Netherlands retrospective study, 544 cases with CU and angioedema identified the mean age at presentation to be 35 years, and patients had been symptomatic an average of 5 years [201].

A prospective study published in 2004 found that duration of urticaria was longer in patients who had associated angioedema or positive anti-IgE receptor antibody [202]. Disease duration is likely to be longer in cases of angioedema, a combination with physical urticaria, positivity in the ASST (autoreactivity), and a high disease severity [6,203].

Malignancy has been linked with urticaria and may suggest a relapse of the malignancy. There is no strong evidence to confirm an association between malignancy and uncomplicated CU, except occasionally in urticarial vasculitis and, more frequently, in acquired C1 esterase inhibitor deficiency [204,205]. Although mortality may occur because of laryngeal edema, death is more likely due to complications of the associated disorder [206].

### Angioedema

In cases involving recurrent angioedema without urticaria, hereditary and acquired angioedema must be differentiated. Acquired angioedema includes, among other etiologies, ACE inhibitor-induced angioedema and angioedema due to acquired C1-inhibitor deficiency. Much like CU, the majority of cases involving acquired angioedema, with some exceptions such as ACE-inhibitor angioedema, can be adequately controlled with daily doses of nonsedating antihistamines [207]. Angioedema of the upper airway can be life threatening. In rare cases, angioedema may develop into anaphylaxis [208].

In Australia, over an 8-year period, there were 106 deaths associated with anaphylaxis or angioedema. According to this study, there was a continuous increase in the rate of hospital admissions for angioedema (3.0% per year) and urticaria (5.7% per year). The rate of hospitalization for angioedema was highest in persons aged 65 years and older and lowest in children between 5 and 14 years. Although the rate of hospital admissions for angioedema remained relatively constant for most age groups between 1993-1994 and 2004-2005, the rate in persons aged 65 years and older doubled from 10 to 20 per 100,000 population. This represented an average annual increase of 5.6% in the rate of admissions for angioedema in this older age group. For those aged 15 to 34 years, the average annual increase was 4.3%. There was no significant change in the rate of hospital admissions for angioedema in those younger than 15 years or from 35 to 64 years. Among older persons, angioedema is becoming an increasing problem [1].

The prognosis for patients with acquired angioedema associated with C1 inhibitor deficiency is variable and depends on control of the underlying disorder. Even with appropriate treatment of the underlying disease, patients may only temporarily be free of symptoms. In several small studies, patients with acquired angioedema associated with C1 inhibitor deficiency had approximately 20% incidence of non-Hodgkin lymphoma [206].

In summary, the prognosis of urticaria and angioedema is improved with prompt and proper treatment. Using available medications, the condition is usually manageable.

## Urticaria and Angioedema in Children

### Prevalence

There is little published information on the prevalence, diagnosis, or management of urticaria in children. Even in the current EAACI/GA2LEN/EDF/WAO and BSACI guidelines on the diagnosis and management of urticaria,[3,4] the section on paediatrics is small.

A very recent review on urticaria in children was published by Church et al,[209] which compared published studies of prevalence of urticaria in adults and in children and noted that CU in children appears to be less common [210]. In the United Kingdom, the incidence of childhood urticaria was around 3.4%,[188] in Germany 4.4%,[147] and in Denmark about 5.4% [211]. In children, most episodes of urticaria appear to be acute, and CU has been reported to affect only 0.1 to 0.3% of children in the United Kingdom [212]. By comparison, 13% of Thai children with a diagnosis of urticaria have been reported to have CU [213].

### Etiology

For AU, infections appear to play a more significant role in infants [214] and children [215].

#### Food Allergy and Parasite Allergy

In a recent study in 80 children with CU, dietician supervised elimination diets of all candidate food allergies suspected by the history and specific IgE levels did not result in any participants reducing or eliminating their requirement for ongoing antihistamine medication, suggesting that food allergy is not an important etiological factor in CU in childhood [216].

#### Antibodies to the IgE Epsilon Receptor

Three independent studies have shown that CSU in children can be caused by autoreactivity as assessed by use of the ASST [216-218].

#### Food Additives

In 1 pediatric study of children between 3 and 17 years, 12 of 16 (75%) were diagnosed with additive-induced urticaria, occurring mainly in response to coloring agents, preservatives, monosodium glutamate, and sweeteners, in the absence of atopy [219].

#### Infections

Although some authors suggest that urinary tract infections followed by *Chlamydia pneumonia *and *H. pylori *[220] were associated with chronic spontaneous urticaria in children, others believe that chronic infection is unlikely to have a significant role in urticaria in children [188,217]. Wedi et al [221] has suggested that in children recurrent upper respiratory infection, pharyngitis, tonsillitis, and sinusitis with streptococci and staphylococci is associated with CU, and remission of urticarial symptoms has been noted with antibiotic therapy.

#### Other Immune Diseases

Although thyroid autoimmunity occurs as a comorbidity in between 14 and 33% of adults with chronic spontaneous urticaria,[209] it has been reported to be much lower in children (about 4.3%) [222]. A small association between CU and celiac disease was also reported in 5% of children with CU [223].

### Natural History of the Disease

In the cohort of pediatric patients with chronic spontaneous urticaria followed for 3 years by Du Toit et al,[216] no clear predictions of disease remission were established; 25% experienced remission in the 3-year period, and this was unrelated to the presence or absence of associated autoimmunity to the IgE Fc*ε *receptor.

By contrast, 58% of children became free of urticarial symptoms in a study of 94 children, of whom 29 were considered "idiopathic" after 16 months, whereas the remaining 42% continued to have recurrent symptoms [224]. A very recent study by Sahiner et al [225] in 2011 found that recovery was observed in 50% of children at 60 months.

### Treatment

In view of the marked adverse effects on the quality of life, ability to play, and school attendance, treatment is necessary in nearly all children with chronic spontaneous urticaria. CU negatively affects school performance. First-generation antihistamines, although effective, are no longer recommended for the management of children with chronic spontaneous urticaria [209].

Second-generation antihistamines are the treatment of choice. In a study of antihistamine treatment given to infants with atopic dermatitis,[226] continuous treatment with levocetirizine significantly reduced exacerbations of concomitant urticaria in this cohort (5.8% vs 16.2% in a placebo group). A follow-up study with levocetirizine showed a 60% reduction in the number of urticarial episodes [227].

Pediatric suspensions of desloratadine, fexofenadine, rupatadine, and loratadine are available, but pediatric studies on these second-generation H1 antihistamines, which are effective in adult urticaria particularly at standard and higher than standard doses, are still to be performed.

In the follow-up study by Du Toit et al,[216] all children responded well to daily treatment with cetirizine, and very few required a short course of prednisone to control symptoms, irrespective of whether they had autoantibodies to the IgE receptor or not.

There are no pediatric studies on the use of leukotriene receptor antagonists, H2 antihistamines, cyclosporine, or omalizumab for the treatment of urticaria. Experience with cyclosporine in children with severe resistant chronic spontaneous urticaria is similar to that reported in adults. It has been found to be safe and highly effective when indicated [228].

There is no evidence in the literature that children with persistent spontaneous urticaria, who do not go into spontaneous remission within a few years, go on to develop other autoimmune diseases. Long-term follow-up studies (more than 10 years) of urticaria in children are awaited.

## Urticaria and Pregnancy

Urticaria may occur in pregnancy as a result of any of the causes seen in nonpregnant women. In women with preexisting CU, the condition may worsen in some patients and appears to improve in others [229].

### New-Onset Urticaria

Urticaria occurring only in pregnancy is rare, but when it occurs, it suggests that sensitivity to hormones may be the basis of the condition. It may recur with each pregnancy in a predisposed woman. Gestational urticaria must be distinguished from other pruritic dermatoses of pregnancy, such as prurigo of pregnancy, PUPPP, PEP, or autoimmune progesterone dermatitis of pregnancy.

### Prurigo of Pregnancy (Prurigo Gestationis of Besnier)

This condition is relatively common occurring in approximately 1 in 300 pregnancies. Characteristically, it begins in the second or third trimester. Patients usually present with marked excoriations with erythematous nodules or papules on the extensor surfaces of the limbs and the trunk. Usually total remission occurs immediately postpartum. Management is usually with topical corticosteroids [230].

### PUPPP (Pruritic Urticarial Plaques and Papules of Pregnancy) or PEP (Polymorphic Eruption of Pregnancy)

PUPPP occurs in 1 in 160 to 1 in 300 pregnancies and usually presents in the third trimester [231,232]. It is seen most commonly in first pregnancies and with multiple births [233]. It presents typically with erythematous papules within the striae and these spread to extremities but spare the face, palms, and soles. Lesions may coalesce to form urticarial plaques. This condition causes extremely severe pruritus. Most commonly, it resolves within 2 weeks of delivery but may resolve beforehand. Occasionally, it may worsen postpartum. Management consists in relieving the distressing symptoms. Topical steroids and antihistamines are initially used; some patients require systemic corticosteroids because of severe pruritus.

### Autoimmune Progesterone Dermatitis of Pregnancy

This condition is similar to the rare autoimmune progesterone urticaria that occurs in a cyclical pattern in nonpregnant women. In pregnancy, it is characterized by a papulopustular eruption, transient arthritis, eosinophilia, and delayed hypersensitivity to intradermal progesterone. It may be associated with spontaneous abortion [234-236].

### Management

Pregnant women with urticaria should be treated with the least amount of medication possible. Most patients can be treated with H1 antihistamines alone, with occasional short courses of oral glucocorticosteroids (GCS) for severe flares.

Antihistamine treatment is the mainstay of management in urticaria. The intense itch experienced by patients demands relief, whereas soothing baths and emollients offer minor comfort; most patients require symptomatic relief with an antihistamine.

There are no oral antihistamines with a category A listing for pregnancy. Categories are based on the results of animal studies, human data, and whether the use of the drug has a positive risk-benefit ratio in pregnancy. Category "B" drugs possess reassuring animal data, but there are no controlled clinical human trials.

A number of studies [237-240] have evaluated the safety of antihistamines in pregnant women. Most women who require regular antihistamines for control of CU will prefer treatment with second generation, nonsedating drugs.

Chlorpheniramine, loratadine, cetirizine, and levocetirizine have all been assigned category B by the US Food and Drugs Administration. As with any medication use, antihistamines should only be used if clearly needed and when the benefits outweigh the potential risk to the fetus. Use of the lowest dose that gives relief is advisable. There are several thousand reports of chlorpheniramine use in pregnancy with no evidence of increased incidence of congenital abnormality. No rate of increased congenital defects was reported in prospectively collected data from 1769 women exposed to loratadine. Small sample size studies are available for cetirizine, and there is a meta-analysis available for loratadine [237,238]. Hydroxyzine is the only antihistamine specifically contraindicated in pregnancy in the product literature.

The second-generation antihistamines of choice in pregnancy are loratadine 10 mg daily or cetirizine 10 mg daily because there is a body of evidence of their use in pregnancy with reassuring safety profiles [240].

In special cases where a sedative effect is required along with an antihistaminic effect, chlorpheniramine is the first-generation antihistamine of choice. Recommended dosing is 4 mg 3 to 4 times a day. Diphenhydramine shows higher efficacy and can be used as an alternative to chlorpheniramine if the use of a first-generation antihistamine is being considered.

### Antihistamines and Breast-feeding

Significant amounts of some antihistamines are detected in breast milk. Again, antihistamines should only be used during lactation when the benefit outweighs the potential harm to the infant, and in this circumstance, use the lowest dose possible for the shortest duration to give relief from symptoms. Loratadine and cetirizine appear safer than others with very low levels recorded in breast milk [241,242].

### Corticosteroids

Systemic corticosteroids may be required periodically to gain temporary control of symptoms during severe exacerbations of urticaria that significantly impair the quality of life. These rescue courses are generally added to the medications the patient is already taking.

The optimal dose and duration of GCS used for urticarial exacerbations has not been systematically studied, and recommendations vary among specialists. In addition, patients differ in their responsiveness to GCS in both the dose and duration of treatment required to control symptoms. Because of their importance in the treatment of a variety of inflammatory conditions, systemic GCS have been used fairly extensively during pregnancy.

Three potential areas of concern have been raised: congenital malformations (primarily cleft palate), neonatal adrenal insufficiency, and low birth weight [243]. The combined results of 5 large studies (2 surveillance and 3 case-control studies) found that the risk of oral clefts is approximately doubled [244-248]. However, the absolute risk is low. Because palatal closure is usually complete by the 12th week of pregnancy, the risk is limited to administration during the first trimester.

Neonatal adrenal insufficiency following maternal administration of steroids is unusual. The rapid maternal metabolism of prednisolone binding to serum proteins and conversion to inactive metabolites by placental 11 beta-hydroxysteroid dehydrogenase results in relatively low fetal compared with maternal concentrations [249]. As a result, the fetal pituitary is rarely suppressed in mothers taking GCS [248]. However, long-term high doses will suppress the fetal adrenal glands.

Multiple studies have observed low birth weight in offspring of animals given GCS during pregnancy. However, this association has been rarely reported in humans [243]. It is difficult to draw conclusions regarding the effects of GCS on fetal growth because of variability in the dose, duration, and type of steroid and the confounding effects of the underlying maternal disease on the pregnancy. GCS have the potential for exacerbating pregnancy-induced hypertension, gestational diabetes, and preterm delivery from premature rupture of membranes [250]. Thus, women at risk should be appropriately monitored.

### Corticosteroids and Breast-feeding

Low levels of prednisone and prednisolone can be measured in breast milk. A nursing infant of a mother consuming a daily dose of 80 mg of prednisolone would ingest < 0.1%, which is equivalent to < 10% of endogenous cortisol production [249]. As a result, although it may be reasonable to delay breast-feeding for several hours after ingesting prednisone, it appears to be safe during breast-feeding [251].

## Quality of life and patient-reported outcomes

During the past 20 years, relevant progresses have been made in defining and evaluating PROs, with growing recognition of their importance in health outcomes research. The expression PROs refers to all health-related reports coming from the patient, without involvement or interpretation by a physician or others [252] [ie, health-related quality of life (HRQoL), symptoms, illness perception, satisfaction, well-being, perceived disease control].

PROs have recently gained great attention in clinical research and by regulatory bodies due to their relevance in the overall treatment efficacy assessment [253-255]. A critical aspect in the management of CU is its impact on the patient's daily experience. The classical symptoms (pruritus, wheals, angioedema) may affect sleep and concentration, interfere with life activities, and cause embarrassment. Furthermore, because of the presence of exacerbations, the unpredictability of attacks, the need to take medication, and change habits and lifestyle, CU patients may experience anxiety, tension, and irritability. Although CU represents a problem that interferes with subjective well-being and daily life, its evaluation has usually focused on clinical end points. As recently underlined,[256] the literature data about CU from a subjective viewpoint remain poor, and most available articles consider mainly 2 PROs: HRQoL and symptoms.

### HRQoL and CU

#### HRQoL Assessment

HRQoL in CU has been assessed by generic, dermatologic-specific, and disease-specific tools. Several generic tools have been used to compare HRQoL of CU patients and healthy subjects--Medical Outcomes Study, SF-36,[257,258] World Health Organization QOL-Brief (WHOQoL-BRIEF)[259]--and CU patients and patients with other diseasesd--Nottingham Health Profile [194]. Although generic instruments permit comparison across different health conditions, they are less suitable for the assessment within a specific disease.

The available questionnaires aimed at assessing HRQoL in skin diseases, the Dermatology Quality of Life Index,[260] and the SKINDEX [261] allow comparisons between different dermatological conditions but are not specifically developed for CU.

The Chronic Urticaria Quality of Life Questionnaire is the only validated specific instrument for CSU and was originally developed in Italian,[262] German,[263] Spanish,[264] Polish,[265] and Turkish [266]. Sixteen validated versions are now available.

#### Impact of CU on HRQoL

Available data show that from a subjective viewpoint, CU is more than an annoying disease. CU subjects report lower HRQoL when compared with healthy subjects or with patients suffering from other medical conditions. A pioneer study by O'Donnell et al [168] compared HRQoL of CU subjects and patients suffering from coronary artery disease. Surprisingly, although patients with ischemic heart disease referred more limitation in mobility, CU patients reported more severe sleep problems. Energy, social isolation, and emotional reactions scores showed similar results between the 2 groups.

Furthermore, HRQoL levels in patients with chronic spontaneous urticaria are lower than in healthy subjects and in patients with respiratory allergy [257]. A study by Poon et al [267] focused on the extent and nature of disability extent and nature in different types of urticaria, showing a large variation in HRQoL scores within different urticarial subsets. In particular, subjects with delayed pressure and cholinergic urticaria showed HRQoL impact comparable with severe atopic dermatitis patients and higher than patients with psoriasis, acne, and vitiligo.

More recently, an article by Staubach et al [268] showed that when compared with healthy subjects, CU patients reported markedly reduced HRQoL. This occurred regardless of age, sex, duration of the disease, and the presence or absence of angioedema. The presence and the severity of psychiatric comorbidities were associated with a more pronounced reduction of HRQoL. Recent studies conducted both in the general population and in outpatients in different countries confirmed that CU impacts HRQoL significantly [12,269].

As yet, the effect of treatment on HRQoL of CU patients has been explored only in 11 trials [77,78,97,111,270-276]. The results of these studies, although different in respect to the drug evaluated, study design, population characteristics, and questionnaire used, indicate an improvement in HRQoL after treatment.

#### CU and Symptoms

CU symptoms can be specifically evaluated with the Urticaria Activity Score [277]. This is the unique validated instrument for measuring and monitoring disease activity in CU. The use of Urticaria Activity Score in clinical practice, trials, and therapy effectiveness analyses [4] is recommended by the current EAACI/GA2LEN/EDF guidelines.

### Actions To Be Taken

Although PROs evaluation is relevant for a more global comprehension of a disease and its treatment, the available literature on CU is still poor. The following unexplored areas should be further investigated:

• Other PROs besides HRQoL and symptoms

• CU impact on caregivers and partners

• Impact of treatment on HRQoL by a specific questionnaire

• Relationships among different PROs and between PROs and psychological variables

• Relation of PROs with other clinical measures of health impact.

## Special considerations of physical urticarias

According to the current international EACCI/GA2LEN/EDF/WAO guidelines on urticaria,[4,5] physical urticaria is defined as a special group of urticaria subtypes, where wheals and/or angioedema are elicited by external physical stimuli.

Physical urticaria needs to be distinguished from both spontaneous urticaria and other inducible urticaria types, such as aquagenic urticaria or cholinergic urticaria, where wheal formation is not induced by a physical stimulus. Physical urticarias usually have a chronic course, but patients can be free of symptoms for weeks or months when the physical stimulus is avoidable and avoided. This is a clear-cut difference to chronic spontaneous urticaria. One point of confusion in the past has been between physical urticaria and cholinergic urticaria. Cholinergic urticaria symptoms can be elicited through a hot shower or bath. The underlying mechanism in cholinergic urticaria, however, is not the external stimulus but the increase in body core temperature; cholinergic urticaria can also be elicited by exercise or emotional distress and is, therefore, included in the urticaria subgroup "other inducible urticarias."

Table 1 (see section Definition and Classification) shows a summary of the physical urticaria subtypes and eliciting factors.

### Diagnosis in Physical Urticaria

Although the current international guidelines on the classification, definition, and diagnosis of urticaria give general recommendations, more detailed recommendations for diagnostic testing in physical urticaria are published in the "European guideline definition and diagnostic testing of physical and cholinergic urticariasd--EACCI/GA^2^LEN/EDF/UNEV consensus panel recommendations."[278]

**Figure 1:**
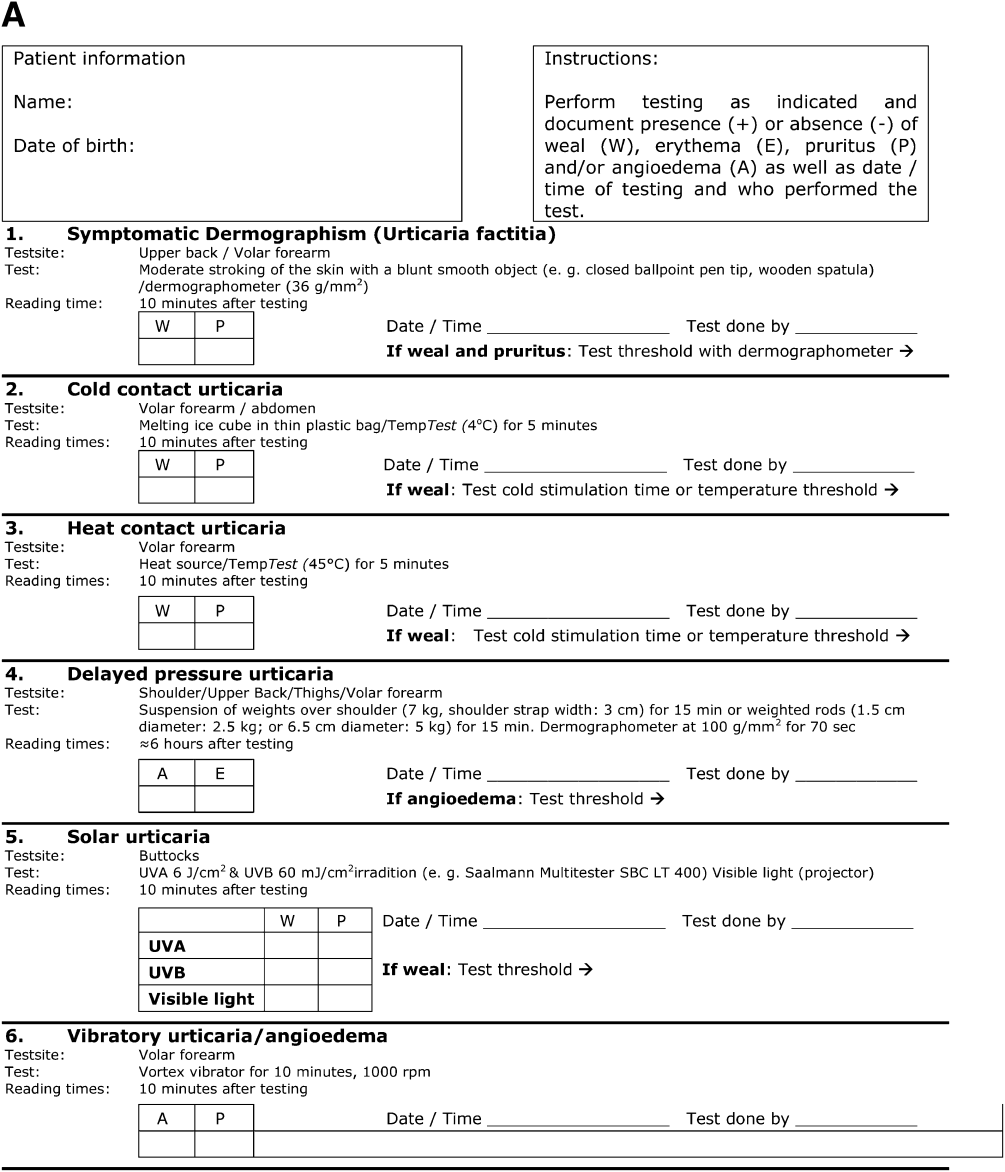
A, Provocation testing for physical and cholinergic urticaria

**Figure 2:**
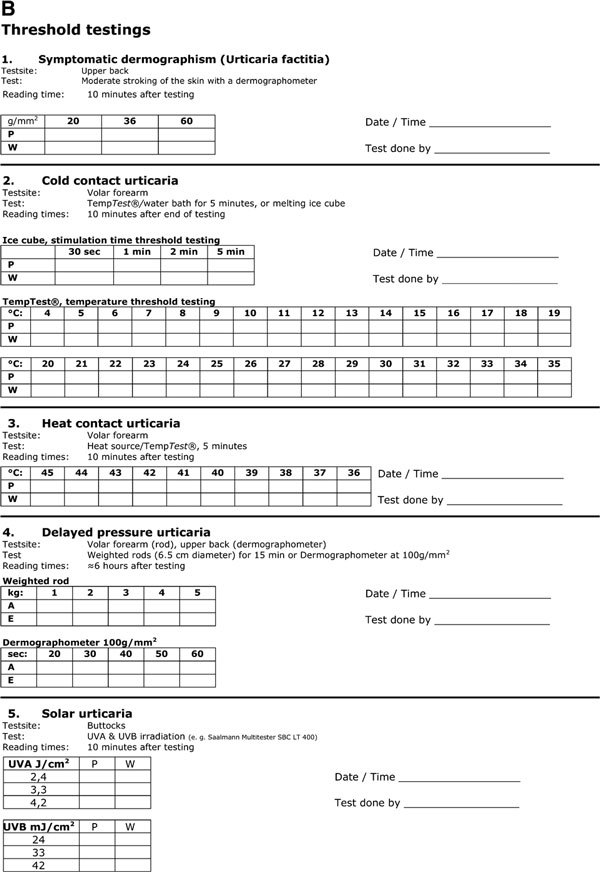
**B, Treshold testing for physical urticaria.**.

A general principle in the diagnosis of physical urticaria is to mimic the physical stimulus, which leads to the formation of wheals and angioedema and at the same time if possible determine the threshold. Threshold measurements are important because they can help to give the patient practical advice on how to avoid or reduce above threshold stimulus exposure. Threshold testing also allows for the objective evaluation and monitoring of patients who receive treatment. Figure 1 and figure 2 show the recommended provocation tests for physical urticaria (modified from Magerl et al [278]).

When performing provocation tests in patients with physical urticaria, it is recommended to have the same standard of emergency treatment available as for other kinds of allergy skin testing because rare cases of systemic anaphylactic reactions, especially in cold urticaria, have been described.

### Severity of Disease and PROs

Physical urticaria can vary considerably in severity between individuals. In a number of patients, signs of physical urticaria only occur with unusually strong external stimulation of the skin, for example, very cold, windy, winter days in cold urticaria, and depending on the usual geographic location and everyday living habits, the required strength of the stimulus to elicit symptoms is not usually reached. However, in other cold urticaria patients, the eliciting temperature of the skin can be as high as 28°C, a temperature which is easily reached in usual daily activities in moderate climates, and even in warm climates, if there is a mild wind because wind chill temperature increases the cooling effect on the skin. In cold urticaria, systemic reactions have been described in the case of a rapid change of skin temperature, for example, when patients jumped into cold water. Another risk factor in cold urticaria is the rapid ingestion of cold food such as ice cream or cold beverages, which may lead to swellings of the upper airways and in the esophagus and to systemic histamine liberation and subsequent anaphylactic reactions.

Physical urticaria can also have an impact on occupation. It has been recognized as an occupational disease (eg, vibratory urticaria/angioedema can be the reason for disability in construction workers).

### Management of Physical Urticaria

A general principle of the international urticaria guidelines on the management of urticaria is the identification and elimination of the underlying cause and/or trigger [5]. Although in the majority of physical urticarias, the underlying cause is unknown and cannot, therefore, be eliminated, avoidance of a known trigger can be very useful.

With the exception of cold contact urticaria where in rare cases infectious diseases, such as hepatitis, have been described as an underlying cause, it is not recommended to invest too many resources into the investigation of causes. In physical urticaria, the routine diagnostic program should be limited at the most to differential blood count and the determination of erythrocyte sedimentation rate. However, with the identification of the eliciting trigger, it is in many cases easy to help the patient by in detail explanation of the possibilities for avoidance. For example, pressure is defined as force per area and simply increasing the size of a handle of a bag may help in patients with pressure urticaria to avoid symptoms.

**Figure 3:**
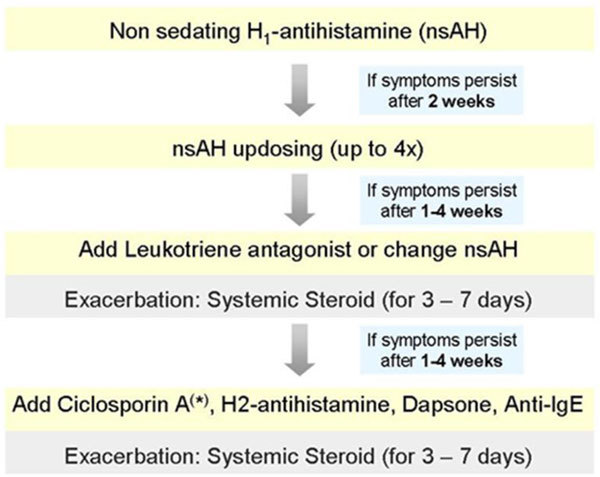
**Algorithm for the treatment of chronic urticaria**.

The treatment in physical urticaria is aimed at the prevention and reduction of symptoms. This follows in general the algorithm, which has been published for urticaria in the international consensus guidelines (Figure 3).

The level of evidence for first-line treatment with nonsedating antihistamines is very good both in chronic spontaneous urticaria and physical urticaria. The updosing of nonsedating antihistamines has been widely studied in physical urticaria.

Thus, the level of evidence to use higher than standard doses as the preferred second-line treatment is very high in this group of urticarias. Siebenhaar et al [279] studied the impact of increasing the dose of desloratadine from 5 mg up to 20 mg in cold urticaria and showed a clear dose-dependent response, which supports the recommendation to increase the antihistamine dosage in those patients who do not show sufficient responses to standard doses.

In general, however, the level of antihistamine treatment required may be different from day to day depending on the strength of the external stimuli and patients' needs to be very thoroughly counseled on the daily use of the drug treatment.

Alternative treatments in physical urticaria have only been scarcely studied and knowledge needs to be extrapolated from what we know from chronic spontaneous urticaria. However, physical urticarias are distinct from other urticaria subtypes in that it is possible to achieve a reduction of repetitive mast cell responses to the specific physical stimulus by long-term controlled exposure to the stimulus. For example, in cold contact urticaria, the occurrence of symptoms can be prevented by administering daily cold baths, and for solar urticaria, UV light treatment can raise UV tolerance. However, this kind of treatment is time consuming for the patient and in the case of cold bath is not always very well liked. Furthermore, it is recommended to start at the threshold level and increase slowly the strength of the physical stimulus because generalized reactions may occur.

## Dissemination and Implementation of the Position Paper

The WAO urticaria and angioedema position paper is being published in the *World Allergy Organization Journal *(WAO Journal) at http://www.WAOJournal.org to facilitate rapid access by all 3000 WAO members. The WAO Member Societies are encouraged to contribute with the dissemination of this position paper through discussion at national and international meetings, and translation and publication in national allergy society journals.

## Summary

This Position Paper presents recommendations for the proper diagnosis and treatment of urticaria and angioedema, highly prevalent diseases in all areas of the world. Although there have recently been important advances in the elucidation of the pathogenesis, allowing the implementation of innovative diagnostic and therapeutic procedures for patients suffering urticaria, the basic mechanisms remain elusive.

Second-generation nonsedating antihistamines at usual or increased doses are presently recommended as first-line therapy for patients with acute and chronic spontaneous urticaria and angioedema. Alternative treatments include H2-antagonists, corticosteroids, leukotriene receptor antagonists, other antiinflammatory drugs, immunosuppressants, omalizumab, and intravenous immunoglobulins.

About one third of patients with CU will continue to experience symptoms after 5 years of follow-up. Consequently, it is important to provide early treatment to improve patient's quality of life. Reduction of the exposure to precipitating and aggravating factors is also important, especially in patients with physical urticarias.

## Note

All authors reviewed and approved this manuscript. *Abstract, Introduction, WAO Global Position Papers, Methods, Prevalence, Dissemination and implementation of the Position Paper, Summary: *M. Sánchez-Borges. *Definition and Classification: *P. Tassinari. *Etiology and Pathogenesis: *A. P. Kaplan and S. Saini. *Diagnostic Approach: *H. S. Park, Y.M. Ye, and I. *Treatment: *A. R. Asero, J. A. Bernstein, D. A. Khan, and A. Tedeschi. *Prognosis: *R. Gower. *Urticaria and Angioedema in Children: *P. Potter. *Urticaria and Pregnancy: *C. Katelaris. *Quality of Life and Patient's Reported Outcomes: *I. Baiardini and G. W. Canonica. *Special Considerations of Physical Urticarias: *M. Maurer and T. Zuberbier.
